# An Investigation of RNA Methylations with Biophysical Approaches in a Cervical Cancer Cell Model

**DOI:** 10.3390/cells13221832

**Published:** 2024-11-06

**Authors:** Buket Sağlam, Onur Akkuş, Azime Akçaöz-Alasar, Çağatay Ceylan, Günnur Güler, Bünyamin Akgül

**Affiliations:** 1 Noncoding RNA Laboratory, Department of Molecular Biology and Genetics, İzmir Institute of Technology, 35430 Izmir, Türkiye; buketsaglam@iyte.edu.tr (B.S.); azimeakcaoz@iyte.edu.tr (A.A.-A.); 2 Biophysics Laboratory, Department of Physics, İzmir Institute of Technology, 35430 Izmir, Türkiye; onurakkus@iyte.edu.tr; 3 Department of Food Engineering, İzmir Institute of Technology, 35430 Izmir, Türkiye; cagatayceylan@iyte.edu.tr

**Keywords:** m^6^A, *N*^6^-methyladenosine, FT-IR, circular dichroism, apoptosis, TNF-α

## Abstract

RNA methylation adds a second layer of genetic information that dictates the post-transcriptional fate of RNAs. Although various methods exist that enable the analysis of RNA methylation in a site-specific or transcriptome-wide manner, whether biophysical approaches can be employed to such analyses is unexplored. In this study, Fourier-transform infrared (FT-IR) and circular dichroism (CD) spectroscopy are employed to examine the methylation status of both synthetic and cellular RNAs. The results show that FT-IR spectroscopy is perfectly capable of quantitatively distinguishing synthetic m^6^A-methylated RNAs from un-methylated ones. Subsequently, FT-IR spectroscopy is successfully employed to assess the changes in the extent of total RNA methylation upon the knockdown of the m^6^A writer, METTL3, in HeLa cells. In addition, the same approach is shown to accurately detect reduction in total RNA methylation upon the treatment of HeLa cells with tumor necrosis factor alpha (TNF-α). It is also demonstrated that m^1^A and m^6^A methylation induce quite a distinct secondary structure on RNAs, as evident from CD spectra. These results strongly suggest that both FT-IR and CD spectroscopy methods can be exploited to uncover biophysical properties impinged on RNAs by methyl moieties, providing a fast, convenient and cheap alternative to the existing methods.

## 1. Introduction

Epitranscriptomics refers to over 170 different types of chemical modifications of ribonucleic acids (RNAs) without any change in their nucleotide sequence, with *N*^6^-methyladenosine (m^6^A) and *N*^1^-methyladenosine (m^1^A) being two of the most frequent types of methylation [1]. RNA methylation is modulated by writers, erasers, and readers specific to each methylation type [2]. For example, methyltransferase 3 (METTL3) and tRNA methyltransferase 61A (TRMT61A) catalyze the deposition of m^6^A and m^1^A, respectively. Since these methylation marks are frequently linked to cellular physiology as well as diseases with a potential for RNA therapeutics [3,4,5,6,7,8,9], several approaches have been developed to measure global, transcriptome-wide, or site-specific changes in m^6^A abundance [10,11]. However, some of these methods are highly labor-intensive and expensive and require highly sophisticated equipment. Additionally, total methylation changes in cells can only be detected by using ELISA-based commercial kits [12], necessitating the need for new, practical, sensitive, and cost-effective techniques to detect changes in RNA methylation.

Fourier-transform infrared (FT-IR) spectroscopy, a vibrational spectroscopic technique, is sensitive to vibrations of molecular bonds present in molecules. It has been successfully applied to obtain detailed (bio)chemical composition and biophysical information about biological samples such as nucleic acids (DNA, RNA), proteins, lipids, cells, tissues or even body fluids [13,14,15,16,17,18]. By following alterations in the intensity and positions of IR signals, FT-IR can be exploited to collect sensitive and rapid data about the biophysical features of RNA, such as conformational changes, dynamics, flexibility, content, and interactions, as well as methylation status. Although FT-IR spectroscopy has been successfully used to evaluate the methylation status of DNA [19,20,21,22], it is unknown if FT-IR can be exploited to assess the presence of RNA methylation marks, such as m^1^A and m^6^A moieties.

Circular dichroism (CD) spectroscopy is an electronic absorption spectroscopy method that measures the absorption difference between the left and right circularly polarized light in the far-UV and near-UV ranges of the electromagnetic spectrum [23,24]. It is a chiro-optical spectroscopic technique widely employed to obtain rapid information (in seconds) on the structural features and interactions of optically active biological macromolecules such as protein, nucleic acids (RNA, DNA), and biopharmaceuticals [14,25,26]. Recently, CD spectroscopy methods have been utilized to inspect changes in the dynamical conformation of nucleic acids in response to various environmental conditions, such as temperature, pH, ionic salt, and interactions with ligand/small organic molecules [17,25,27,28,29]. A typical CD spectrum of RNA exhibits a negative peak around 200–210 nm, attributed to the right-handed (A- and B forms) RNA molecule, and displays a positive CD signal around 260–270 nm arising from base-pairing and base-stacking contributions [29,30,31]. Recently, CD spectroscopy has been used to track conformational changes in synthetic RNA oligos upon demethylation by fat mass and obesity-associated protein (FTO), and AlkB homologue 5 (ALKBH5) [32,33]. These studies demonstrate that RNA m^6^A demethylation is associated with base-stacking alterations and hairpin conformational changes, which can be detected in the CD spectrum [29,32].

In this study, we have exploited FT-IR and CD spectroscopy methods to assess changes in the m^6^A RNA methylation marks, as well as their impact on RNA structures, using both synthetic and cellular RNAs. Our data show that FT-IR can be successfully employed to differentiate m^6^A-methylated synthetic RNA oligonucleotides from un-methylated ones. We have also demonstrated that FT-IR can safely measure perturbations in the global m^6^A RNA marks upon METTL3 knockdown in eukaryotic cells. CD spectroscopy was then used to assess the impact of METTL3 and TRMT61A knockdown on secondary structures of RNAs. Finally, we show that our protocol can also be applied to assess perturbations in methylation marks upon extracellular stimuli in eukaryotic cells, such as tumor necrosis factor α (TNF-α).

## 2. Materials and Methods

### 2.1. Cell Culture, Flow Cytometry and Western Blotting

HeLa cervical cancer cells that were supplied from DKFZ GmbH (Germany) were cultured in RPMI 1640 (with 2 mM L-Glutamine, Gibco, Grand Island, NY, USA) reinforced with 10% fetal bovine serum (FBS) (Gibco, Grand Island, NY, USA) and incubated in a humidified atmosphere of 5% CO_2_ at 37 °C. Three biological replicates of 0.8 × 10^6^ HeLa cells were treated with 37.5 ng/mL TNF-α (Biolegend, San Diego, CA, USA) in the presence of 2.5 µg/mL of cycloheximide (CHX) (Applichem, Darmstadt, Germany) for 24 h to induce apoptosis, as reported previously [4]. CHX treatment was used as the negative control. Cells were trypsinized by 1X Trypsin-EDTA (0.25%) (Gibco, Grand Island, NY, USA), washed by 1X cold PBS (Gibco, Grand Island, NY, USA) and centrifuged at 1000 RPM at room temperature (RT) for 5 min. The cell pellet was dissolved in 1X Annexin binding buffer (Becton Dickinson, Franklin Lake, NJ, USA) and stained with Annexin V-FITC (Biolegend, San Diego, CA, USA) and 7AAD (Biolegend, San Diego, CA, USA), followed by 15 min incubation in the dark. The rate of apoptosis was measured by flow cytometry (BD FACSCanto, Vincent Moraga Drive Temecula, CA, USA).

RIPA buffer (Cell Signaling Technology, Danvers, MA, USA) supplemented with a protease inhibitor cocktail was used to obtain total protein extracts from transfected or treated HeLa cells. Western blotting was performed according to a previously published procedure [4]. Primary antibodies included METTL3 (Rabbit #96391, Cell Signaling Technology, Danvers, MA, USA), TRMT61A (Rabbit #PA5-76553 ThermoFisher Scientific, Waltham, MA, USA), Caspase 8 (Mouse mAb #9746, Cell Signaling Technology, Danvers, MA, USA), Caspase 3 (Rabbit mAb #14220, Cell Signaling Technology, Danvers, MA, USA), and ß-actin (Rabbit mAb #4970, CST). ß-actin was used as an internal control. Band intensities were quantitated using ImageJ 2.9.0/1.53t/Java 1.8.0_322.

### 2.2. Cell Transfection

A siRNA pool specifically designed to knockdown METTL3 (si-METTL3), TRMT61A (si-TRMT61A) and its negative control non-target siRNA (si-NC) were obtained from Dharmacon (Lafayette, CO, USA). Briefly, 0.6 × 10^6^ HeLa cells were plated on 10 cm dishes (Sarstedt, Germany) and transfected with 25 nM siRNA pool after overnight incubation, as previously described [4]. Cells were typically incubated post transfection for 72 h unless indicated otherwise.

### 2.3. Total RNA Isolation and Global m^6^A Detection

Total RNAs were isolated from treated or transfected cells using GeneAll^®^ RiboExTM reagent (GeneAll Biotechnology Co., Seoul, Republic of Korea) according to the manufacturer’s instructions. The Invitrogen™ TURBO DNA-free™ kit (Thermo Fisher Scientific, Waltham, MA, USA) was used to remove remaining genomic DNA from RNA samples according to the manufacturer’s instructions. RNAs were stored at −80 °C until use. The colorimetric epiquick m^6^A RNA methylation quantification kit (Epigentek, New York, NY, USA) was used to measure m^6^A methylation levels according to the manufacturer’s protocol.

### 2.4. SELECT (Single-Base Elongation- and Ligation-Based qPCR Amplification Method)

The site-specific changes in m^6^A marks were assessed according to a previously published protocol [34]. The SELECT protocol is composed of two different PCR reactions and a qRT-PCR reaction. Firstly, 1500 ng of RNA was adjusted for each reaction and filled up to 12 µL with distilled water. A total of 2 µL of 10X CutSmart Buffer [CSB; (50 mM KAc, 20 mM Tris-HAc, 10 mM MgAc2, 100 μg/mL BSA, pH 7.9 at 25 °C)], 1 µL of 5 µM dTTP, 1 µL of 40 nM forward, and 1 µL of 40 nM reverse oligos were added to each well for the first PCR reaction, which was set to 90 °C for 1 min, 80 °C for 1 min, 70 °C for 1 min, 60 °C for 1 min, 50 °C for 1 min and 40 °C for 6 min. Then, 1 μL of 0.01 U Bst 2.0 polymerase, 1 μL of 0.5 U Splint R ligase diluted in Diluent A, and 1 μL of 10 mM ATP were added to the resulting mixture. A total volume of 20 μL reaction mixture was used for the second PCR reaction, with the settings of 40 °C for 20 min and 80 °C for 20 min. Subsequently, qRT-PCR analysis was performed by using 0.5 µL of 4 µM SELECT primer and 1 µL of final mixture with the aid of Rotor-Gene Q machine (Qiagen, Hilden, Germany). Ampliqon RealQ Plus 2x Master Mix Green (Odense, Denmark) was used for qRT-PCR (95 °C for 15 min holding, 95 °C for 20 s, 60 °C for 60 s and 40 times cycling). The oligomers and primers are listed in Table 1. An un-methylated site of MALAT1 lncRNA was used as negative control.

### 2.5. FT-IR Spectroscopy Measurements

FT-IR (Perkin Elmer, Waltham, MA, USA, UATR Two) combined with an attenuated total reflection (ATR) unit and equipped with a MIR TGS (Mid-infrared Triglycine Sulfate) were used to analyze synthetic and cellular RNAs. The FT-IR spectra were recorded with 32 co-added scans at a 2 cm^−1^ spectral resolution in the wavenumber range of 4000–800 cm^−1^ at RT. When the ATR crystal was clean, the air spectrum was recorded as background. Three replicates of RNA samples were analyzed at a concentration of 2 μg/µL and 2.5 μg/µL for synthetic (Eurofins Genomics, Ebersberg, Germany) and total RNAs, respectively. For each replicate measurement, only 2 µL of RNA sample (a very small droplet) was placed on the ATR diamond crystal and gently dried at RT for about 8 min under dry-air purge conditions to eliminate water excess. The drying of the sample was tracked by monitoring the OH stretching mode (4000–3000 cm^−1^). For each replica measurement, at least four spectra were recorded after the drying of RNA samples on the ATR unit. The last 4 spectra of each replicate were averaged and used for further analysis. After each measurement, the ATR unit was cleaned with propanol and copious distilled water. For cell-derived RNA samples, three biologic replicates originate from different independent cultures and their three technique replicates were measured.

To prepare the synthetic RNA mixtures at various concentrations of m^6^A methylation, 2 μg of m^6^A synthetic RNAs (so-called 100% methylated or 100% m^6^A RNA) and 2 μg of un-methylated synthetic RNAs (so-called 0% methylated or 0% m^6^A RNA) were mixed at appropriate amounts of volume (*v*/*v*). Thus, the ratio of synthetic m^6^A RNAs (100% methylation) to un-methylated synthetic RNAs (0% methylation) were prepared as follows: 0:4, 1:3, 1:1, 3:1 and 4:0 (0%, 25%, 50%, 75% and 100% methylation, respectively).

For FT-IR data analysis and visualization, the ‘OPUS 7.0’ (Bruker, Bremen, Germany), OriginPro 2024 and ‘Kinetics’ software running under MATLAB (R2011b) were employed, as described previously [13,14,15]. Accordingly, each absorbance spectrum was baseline-corrected by interpolating straight lines between the points of the spectrum at multiple wavenumbers (3970 cm^−1^, 3715 cm^−1^, 2800 cm^−1^, 2500 cm^−1^, 1800 cm^−1^, 1750 cm^−1^, 1510 cm^−1^, 1438 cm^−1^, 1315 cm^−1^, 946 cm^−1^, 895 cm^−1^, 835 cm^−1^, and 801 cm^−1^) and they were subtracted from the corresponding spectrum. Later on, those spectra were normalized for an equal area between 1750 cm^−1^ and 1513 cm^−1^, as this range is not dependent on the methylation signals (CH_3_). Thus, the alterations in the methylation levels of RNA samples were described based on a constant amount of nitrogenous bases. Principally, the IR signals acquired from different RNA groups need to be normalized to an internal reference or total intensity, such as for the same amount of bases, since relative intensities are used for comparison. Thereby, the normalization of the FT-IR spectra helps to eliminate instrumental differences and/or concentration-dependent variations at each time of data acquisition. In the current study, thus, the absorbance scale of FT-IR spectra represents re-scaled absorbance values in the figures. Finally, those fully pre-processed (baseline-corrected and normalized) spectra were used for the assessment of RNA methylation levels. The FT-IR 2nd derivative spectrum was then calculated from the average absorbance spectrum for each RNA group using the Savitzky–Golay algorithm with 9 smoothing points to resolve the superimposed bands. The order of the aforementioned data analysis procedure (from raw spectra to fully pre-processed spectra) was shown in Supplementary Figure S1 point-by-point as an explicit example of this data reduction. Accordingly, it is clear that the spectral changes due to presence of RNA methylation are independent from this data processing.

The ‘average’ absorbance spectrum calculated for each synthetic RNA sample were used in the calculation of band intensities at 2984 cm^−1^, 2883 cm^−1^, 1478 cm^−1^ and at 1363 cm^−1^ by using the R-method in OPUS 7.0 software program (Bruker, Germany). Herein, first, a straight baseline was drawn between the peaks of the two frequency limits defined (for 2984 cm^−1^ and 2883 cm^−1^: 3012–2865 cm^−1^, for 1478 cm^−1^: 1513–1437 cm^−1^ and for 1363 cm^−1^: 1436–1344 cm^−1^), and then, the intensity value above that line for each peak was read. Subsequently, those peak intensities were plotted against m^6^A methylation concentrations (from 0% to 100%) to determine the correlation R^2^ value (which provides knowledge on the goodness of fit of a model) between the methylation concentrations and CH_3_ signals.

### 2.6. CD Spectroscopy Measurements

The CD spectra of RNA samples were recorded by using a JASCO J-1500 CD spectrometer (Jasco, Tokyo, Japan) at RT. CD spectrum measurements were performed with synthetic and total cellular RNAs at a final concentration of 114 and 26.8 ng/µL, respectively, suspended in 10 mM NaPi pH 7.4 150 mM NaCl with RNAse free water. Each RNA sample with a volume of 500 μL was placed in a quartz cuvette (d = 0.2 cm pathlength), and the CD spectra were recorded in triplicates in the range of 190–350 nm with the parameters, as follows: Bandwidth 1.0 nm, Data pitch: 0.1 nm, DIT: 4 s, number of accumulation scans: 8, scanning speed: 50 nm/min.

CD data analysis and visualization were performed with ‘OPUS 7.0’ (Bruker, Germany) and OriginPro 2024, as described previously [14,26]. The 1st derivative of the CD spectra was also computed using the Savitzky–Golay algorithm with 9 smoothing points to resolve the superimposed bands. No further treatments were carried out for CD data.

## 3. Results

### 3.1. m^6^A Methylation Induces a Unique Spectroscopy Profile in Synthetic RNA Oligonucleotides

**Figure 1:**
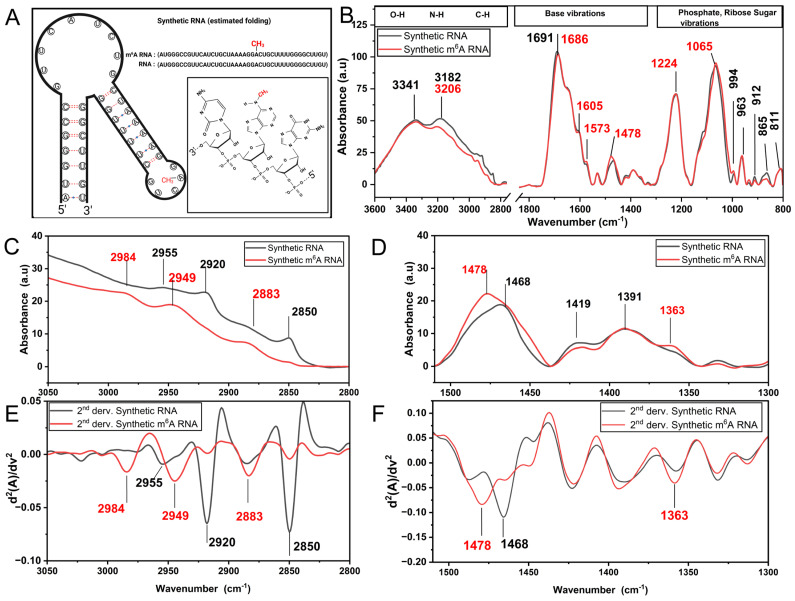
The FT-IR average spectrum of synthetic RNA samples (both at 2 μg/µL concentration). (**A**) The sequence of synthetic RNAs and their estimated secondary structure. (**B**) FT-IR absorbance spectra in the 3600–800 cm^−1^ spectral range. A magnification the FT-IR (**C**,**D**) absorbance spectra and (**E**,**F**) second derivative spectra are also shown, representing the CH stretching and bending vibrations, respectively. The synthetic RNA samples without methylation (black) and with m^6^A methylation (red) are displayed. The peaks are color coded.

FT-IR spectroscopy has been widely used to assess the biophysical properties of proteins and, to a lesser extent, RNAs [35,36,37]. RNA methylation is known to cause structural switches and expose protein binding sites [38,39]. We hypothesized that RNA methylation-modulated alterations in the RNA folding landscape and exposure of the m^6^A moiety out of the helix [40] might facilitate the easy detection of RNA methylation in the FT-IR spectra. To test the proof of concept, we first aimed to compare the FT-IR profiles of two RNA oligonucleotides, one with and the other without an m^6^A mark. To this extent, as a positive control, we selected a 30-nucleotide RNA oligonucleotide, which was previously used as a bait to investigate m^6^A:reader interaction (Figure 1A) [41]. The FT-IR spectrum of un-methylated and m^6^A-methylated synthetic RNA samples (Figure 1B) contains three main fingerprint regions originated from the vibrations of RNA functional groups, as follows: (i) stretching vibrational modes of the O-H, N-H, and C-H groups (3500–2800 cm^−1^), (ii) nitrogenous base vibrations (1800–1500 cm^−1^), and (iii) vibrations of phosphate groups and ribose sugar groups (1300–800 cm^−1^) (for band assignments see refs. [16,17,37,42,43]). In our study, since the RNA samples were suspended in RNAse free water, there is no spectral contribution arising from solution of RNA. Additionally, RNA samples were gently dried (so-called dehydrated RNA) during FT-IR measurements in order to avoid hydration-dependent spectral contributions in the RNA spectra. Thus, the spectra of dehydrated RNA molecules include only bonded OH groups present intrinsically in the RNA molecule but not free water molecules. Herein, we applied the same drying procedure for all RNA groups, which is not uncommon, so that we can compare different samples for assessment. Many biological samples (cells, RNA, DNA, body fluids, etc.) are mostly analyzed with FT-IR under dry conditions [13,16,19,44].

In fact, IR signals resulting from the vibrations of CH_3_ groups (methyl moiety) are expected to appear due to methylation [14,19]. The functional groups of RNA nucleotides do not possess any natural methyl moieties in their natural structure (but thymine has CH_3_ in DNA). Inspired by this information, the IR bands comprising CH_3_ group vibrations were analyzed in detail (Figure 1C,D). When compared to the un-methylated synthetic RNA samples, the m^6^A-methylated synthetic RNAs exhibit substantial spectral differences particularly in the IR regions of 3000–2800 cm^−1^ (CH stretching vibrations) and 1500–1350 cm^−1^ (CH bending vibrations). We detected intense absorption bands with maximum values at 1478 cm^−1^ and 1363 cm^−1^ upon m^6^A methylation, which are due to the asymmetric and symmetric bending vibrations of CH_3_ groups (methyl moiety), respectively. Additionally, m^6^A-methylated synthetic RNAs give rise to pronounced IR peaks absorbing at 2984 cm^−1^ and 2883 cm^−1^ due to the asymmetric and symmetric stretching vibrations of methyl moiety, respectively. This is accompanied by contributions of weak IR peaks at 2949 cm^−1^ and 2850 cm^−1^, but they arise from the asymmetric and symmetric stretching vibrations of CH_2_ groups in RNA functional groups, respectively (not due to the methyl moiety). Those spectral differences are resolved better in the FT-IR second derivative spectra, representing the minima of the peaks (Figure 1E,F). However, un-methylated synthetic RNAs exhibit absorption bands located at 1468 cm^−1^ due to the bending vibrations of CH_2_ groups present in ribose sugar and bases, and exhibit IR signals absorbing at 2955 cm^−1^ and 2920 cm^−1^ and at 2850 cm^−1^ due to the asymmetric and symmetric stretching vibrations of CH_2_ groups, respectively. Evidently, major spectral differences exist between the methylated and un-methylated synthetic RNA samples, stemming from the methyl moiety.

Apart from the signatures of methyl moiety, FT-IR spectral differences are hardly observed in the range of 3500–3100 cm^−1^ (O-H, N-H groups), 1800–1500 cm^−1^ (bases), and 1300–800 cm^−1^ (sugar-phosphate backbone) (Figure 1B). In the case of methylated synthetic RNA samples, the intensity in the spectral range of 3200–3100 cm^−1^ (stretching vibrations of N-H groups) is reduced, in concomitant with a small increment in the intensities of the peaks at 1605 cm^−1^ and 1573 cm^−1^ (due to in-plane ring vibrations of adenine). This is most likely due to the attachment of a methyl moiety to the NH_2_ group at the *N*^6^ position of adenosine nucleotide, forming the NH group in m^6^A RNA methylation, and affecting the adenine molecular vibrations. Moreover, the IR bands in the range of 1800–1500 cm^−1^, arising from the base vibrations (C=C, C=N and C=O stretching), exhibit only slight differences. The peak absorbing at 1691 cm^−1^ is shifted down towards 1686 cm^−1^ (C=O stretching vibrations of guanine), reflecting an indirect effect of methylation. These altogether strongly demonstrate that the changes in the position and/or intensity of those bands reflect only slight alterations in the microenvironment and/or base-pairing/base-stacking properties due to m^6^A methylation. Spectral variations are also noticed for phosphates moieties of RNA backbone. The intensities of the peaks absorbing at 1224 cm^−1^ and 1065 cm^−1^ (antisymmetric and symmetric stretching vibrations of (PO2)−, respectively) increase slightly for m^6^A RNA methylated synthetic samples (Figure 1B). In the spectral range of sugar backbone vibrations, three characteristic IR peaks absorbing at 880 cm^−1^, 865 cm^−1^ and 811 cm^−1^ can be observed arising from the N-type (C3′-endo) ribose sugar, corresponding to A form of RNA most likely due to its nature, rather than the dehydration process (please see the CD results). The intensities of the peaks at 912 cm^−1^ (ribose ring vibration) and 865 cm^−1^ (N-type sugar) decrease slightly as well, reflecting conformational changes in the sugar backbone of synthetic RNA upon m^6^A methylation (for band assignments see refs. [17,19,37,42,43]).

**Figure 2:**
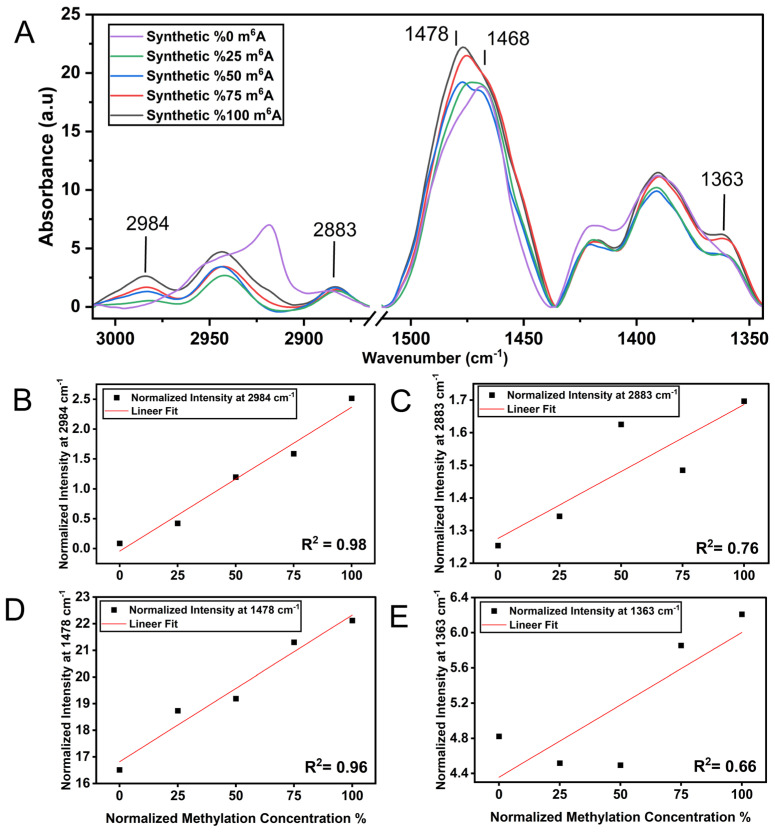
The FT-IR spectrum of mixtures of synthetic RNA samples. (**A**) The FT-IR average spectra of synthetic RNA (0% methylation), m^6^A RNA (100% methylation) and their mixtures with the ratio of 1:3, 1:1, 3:1 (25%, 50%, 75% methylation, respectively), showing the spectral region of stretching and bending vibrations of CH molecules. Methylation concentration was plotted versus the intensities (**B**) at 2984 cm^−1^ with R^2^: 0.98, (**C**) at 2883 cm^−1^ with R^2^: 0.76, (**D**) at 1478 cm^−1^ with R^2^: 0.96 and (**E**) at 1363 cm^−1^ with R^2^: 0.66. To prepare the RNA mixtures at various ratios, 2 μg of m^6^A synthetic RNAs (100% methylated) and 2 μg of un-methylated synthetic RNAs (so-called 0% methylated) were mixed at appropriate amounts of volume.

**Figure 3:**
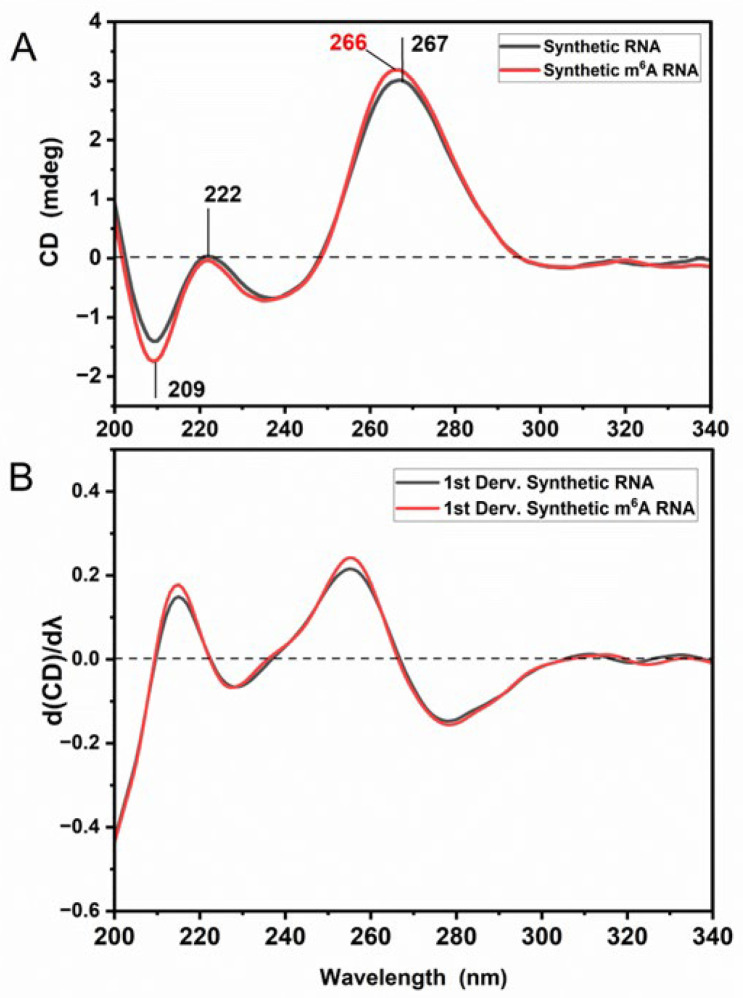
The CD spectra of synthetic RNA samples (both at 26.8 ng/µL concentration). (**A**) The CD absorbance spectra and (**B**) its 1st derivative spectra without methylation (black) and with m^6^A methylation (red) are displayed. The peaks are color coded.

Although the qualitative measurement of global changes in RNA m^6^A methylation by FT-IR could be of interest, it would be more valuable to be able to interrogate the extent of methylation quantitatively. To this extent, we mixed synthetic un-methylated (so-called 0% methylated) and m^6^A RNA samples (so-called 100% methylated) in different combinations (*v*/*v*), ranging from 0% to 100% methylation and assessed the ability of FT-IR to quantitatively predict the percentage of m^6^A methylation in RNA samples. As shown in Figure 2A, when the m^6^A methylation rate increases in RNA samples from 0% to 100%, the relative intensities at 2984 cm^−1^, 2883 cm^−1^, 1363 cm^−1^ and at 1478 cm^−1^ (due to vibrations of CH_3_ groups) increase simultaneously. The latter is upshifted from 1468 cm^−1^ (CH_2_ groups) to 1478 cm^−1^ (CH_3_ groups) as well. More interestingly, we observed a linear fit proportional to the percentage of RNA methylation for both intensity values at 2984 cm^−1^ (R^2^: 0.98) and 1478 cm^−1^ (R^2^: 0.96) (Figure 2B–D), clearly demonstrating the high correlations between RNA methylation and intensities at both 2984 cm^−1^ and 1478 cm^−1^. Nevertheless, the peak at 2984 cm^−1^ might be affected from the spectral contributions of other intrinsic molecular groups, such as O-H and N-H stretching vibrations detected close to the CH_3_ stretching vibrations in the spectral region of 3000–2800 cm^−1^; thus, a dehydration protocol should be carefully followed during measurements. The methylation concentration was also plotted against the intensities at 1363 cm^−1^ (R^2^: 0.66) and at 2883 cm^−1^ (R^2^: 0.76) (Figure 2C–E), but their correlations were not as strong. This altogether strongly indicates that the FT-IR spectral range of CH_3_ stretching and bending vibrations, particularly the intensities at 2984 cm^−1^ and 1478 cm^−1^, can be successfully used as intrinsic markers without labeling for the quantification of RNA methylation.

m^6^A marks are likely to affect the RNA structure [38,39]. Thus, we examined the CD spectral pattern of synthetic RNAs, which is based on the stacking geometry of the bases (primarily) and the intrinsic asymmetry of functional groups (chiral sugar groups, phosphate groups and bases) [31]. The CD signals in the spectral range of 200–300 nm correspond to the interactions of π-π * oscillations and n-π * transitions between bases and the sugar-phosphate backbone in the RNA molecule. The CD spectra of un-methylated synthetic RNA oligonucleotides display characteristics CD signals, harboring a negative peak around 209 nm and two positive peaks at 267 nm (strong) and 222 nm (weak) (Figure 3A). The positive band absorbing at 267 nm is under the effect of intra-molecular and inter-molecular base-stacking and base-pairing features. The CD spectrum, exhibiting a maximum at 267 nm, a minimum near 209 nm, and a weak negative CD signal between 290 and 300 nm, reflects the forming of right-handed stack in A-form RNA conformation. The CD measurements of RNA were performed in aqueous solutions; thus, it is not due to the dehydration process. Moreover, the detection of a positive CD signal at 222 nm is related to the helical twisting of the RNA structure (for band assignments, see refs. [24,31,45]). The CD spectral profile of m6A-methylated synthetic RNA exhibits alterations in the peak amplitudes and wavelength positions. Accordingly, the amplitude of the negative peak at 209 nm increases becoming more negative and the positive peak at 267 nm shifts down towards 266 nm, concomitant with an increment in its CD amplitude. Those spectral differences are also resolved in the CD 1st derivative spectra (Figure 3B). Based on the CD data, synthetic m^6^A RNA oligonucleotides probed here undergo alterations in their chirality and base-stacking features due to the presence of a methyl group.

### 3.2. Spectroscopic Analysis of Perturbations in Cellular m6A Marks

**Figure 4:**
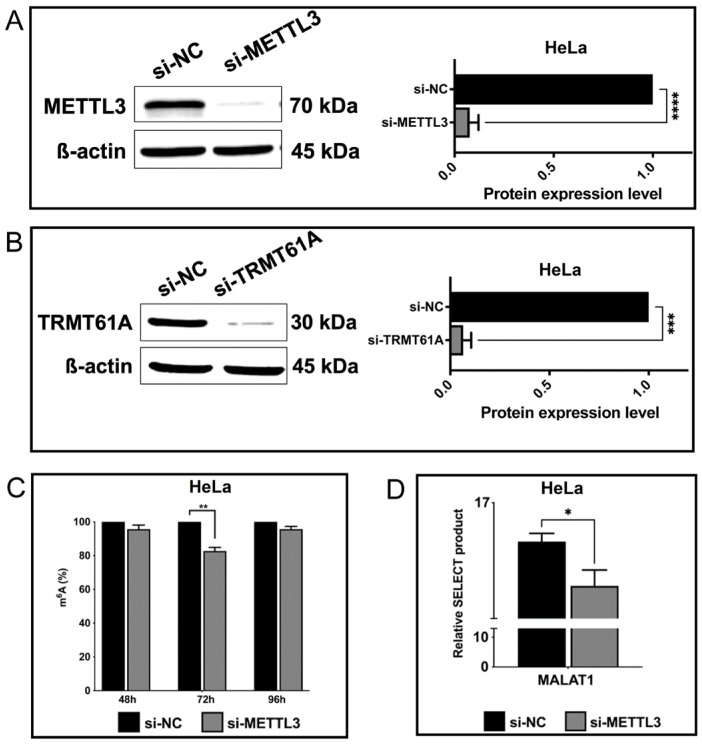
The effects of the knockdown of methylation writers on cellular m^6^A status. (**A**) Western blotting results to verify METTL3 knockdown in HeLa cells (fold change). (**B**) Western blot results of TRMT61A knocked-down HeLa cells (fold change). (**C**) The percentage change in m^6^A modification in the total RNA after METTL3 knockdown in HeLa cells (N = 2 for all time points) (**D**) SELECT results for METTL3 knocked-down HeLa cells by operating a site-specific detection of m^6^A modification on the MALAT1 gene. Data were normalized by MALAT1 A2511. Error bars indicate mean ± SD of three biological replicates with two technical replicates, unless indicated otherwise. The statistical significance was demonstrated as * *p* < 0.05; ** *p* < 0.01; *** *p* < 0.001; **** *p* < 0.0001 by two-tailed Student’s *t* test.

**Figure 5:**
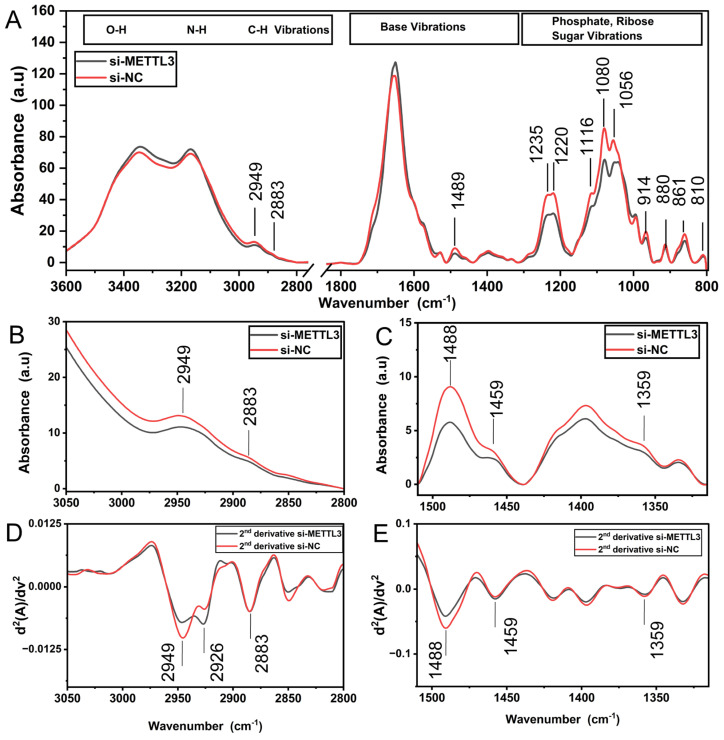
The FT-IR average spectra of cell-derived RNA samples from HeLa si-METTL3 and HeLa si-NC (both at 2.5 μg/µL concentration). (**A**) FT-IR absorbance spectra in the 3600–800 cm^−1^ spectral range. (**B**,**C**) FT-IR absorbance spectra and (**D**,**E**) second derivative spectra, representing the CH stretching and bending vibrations. The IR peaks for RNA samples of HeLa si-METTL3 (black) and HeLa si-NC (red) are color coded.

Since FT-IR can successfully differentiate methylated synthetic RNAs from un-methylated ones (Figure 1B), we interrogated whether it could be employed to assess the extent of m^6^A and m^1^A methylation in cellular RNAs. To this end, we transfected HeLa cells with siRNA pools to knock down METTL3, the main catalytic enzyme in the m^6^A writer complex, and TRMT61A, the main enzyme in the m^1^A writer complex [46,47]. The use of a siRNA pool ensured the efficient knockdown of the target METTL3 protein without any off-target effect as the pool contains several siRNAs at relatively much lower concentrations (Dharmacon, CO, USA). The Western blot analyses showed that the siRNA pool was able to reduce METTL3 and TRMT61A amounts by 94.78 and 93.53%, respectively (Figure 4A,B). We then employed a colorimetric detection kit to assess the extent of reduction in the percentage of m^6^A-methylated RNAs upon METTL3 knockdown. As shown in Figure 4C, si-METTL3-transfected HeLa cells displayed 18.78% reduction in m^6^A-methylated RNAs, further supporting the efficient knockdown of METTL3. Additionally, we assessed the extent of methylation at 2515th adenine residue in metastasis-associated lung adenocarcinoma transcript (MALAT) long noncoding RNA (lncRNA) upon METTL3 knockdown. Our data showed that METTL3 knockdown led to a 59% reduction in the proportion of m^6^A marks at this site (Figure 4D). We then compared the FTIR profile of total RNAs isolated from HeLa cells transfected with si-NC or si-METTL3 (Figure 4). Overall, the FT-IR spectrum of cellular total RNA samples (Figure 5A) moderately resembles that of synthetic RNA oligonucleotides (Figure 1B), representing strong/weak IR bands at quite similar positions due to the contributions of RNA functional groups (nitrogenous bases, phosphate-sugar backbone). Nevertheless, cellular total RNAs isolated from control and METTL3 knockdown HeLa cells exhibit substantial spectral differences in the mid-IR range of 3500–2800 cm^−1^ (stretching vibrations of O-H, N-H, C-H groups), 1800–1550 cm^−1^ (base vibrations), and 1300–800 cm^−1^ (vibrations of phosphate-sugar backbone) (for band assignments, see refs. [17,37,42,43,48]). The spectrum of RNA molecules of METTL3 knockdown cells exhibits less intense IR bands in the range of 3000–2800 cm^−1^ and 1500–1350 cm^−1^, attributed to the stretching and bending vibrational range for CH_3_ groups, respectively, in comparison to the total RNAs of control HeLa cells. Accordingly, the intensities of IR signals that appear at 2949 cm^−1^ (broad band) and 2883 cm^−1^ (asymmetric and symmetric stretching vibrations of CH_3_ groups, respectively) and at 1488 cm^−1^ and 1359 cm^−1^ (the asymmetric and symmetric bending vibrations of CH_3_ groups, respectively) are dramatically reduced upon METTL3 knockdown cells (Figure 5B,C). Those IR signals (2949 cm^−1^, 2883 cm^−1^, 1488 cm^−1^ and 1359 cm^−1^) are the characteristic markers for methylation involved in the RNA molecule and are resolved better in the FT-IR second derivative spectra, showing the minima of those peaks (Figure 5D,E). Thus, this spectral pattern strongly reveals a reduction in the CH_3_ signals of methyl groups upon METTL3 knockdown (for band assignments see refs. [19,49,50]). Such IR bands due to methyl moiety are also detected in the case of synthetic RNA oligonucleotides upon methylation (Figure 1).

The spectral profiles of control and METTL3 knockdown HeLa cells also have significant differences in characteristic peaks attributed to the molecular vibrations of functional groups (base, sugar, and phosphate) (Figure 5A). In comparison to the control group, the FT-IR spectrum of METTL3 knockdown RNA samples exhibits slightly more intense bands in the spectral range of 3500–3000 cm^−1^ (stretching vibrations of O-H and N-H groups) and of 1715–1550 cm^−1^ (base vibrations) but exhibits less intense IR bands at 1235–1220 cm^−1^ and 1080 cm^−1^ (the asymmetric and symmetric stretching vibrations of phosphate groups, respectively) and at around 1056 cm^−1^ accompanied by a peak at 1116 cm^−1^ (stretching vibrations of ribose sugar). Moreover, spectral differences are also noticed in the lower spectral region of sugar backbone vibrations. The detection of the three distinct IR peaks absorbing at 880 cm^−1^, 861 cm^−1^ and 810 cm^−1^ are attributed to the N-type (C3′-endo) ribose sugar, corresponding to the A form of RNA. The intensities of the IR bands appearing at 914 cm^−1^ (ribose ring vibration) and 861 cm^−1^ (N-type sugar) are reduced as well, reflecting conformational changes in the sugar backbone upon METTL3 knockdown (for band assignments see refs. [16,17,37,42,43]). To sum up, the vibrations of N-H groups, nitrogenous bases, and the phosphate-sugar backbone of RNA exhibit alterations upon METTL3 knockdown, and the IR peaks arising from CH_3_ vibrations are unequivocal indicators for the existence of methylation in RNA.

**Figure 6:**
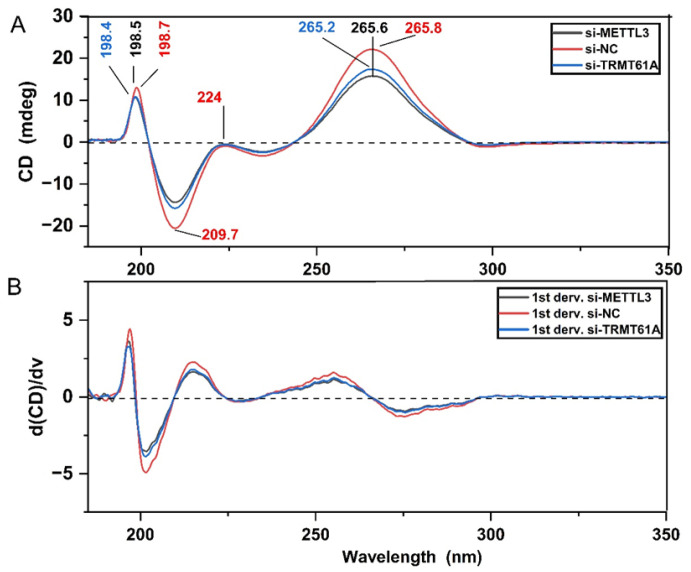
The CD spectra of RNA samples (all at 114 ng/µL concentration) isolated from HeLa cells (HeLa si-NC, HeLa si-METTL3, HeLa si-TRMT61A). (**A**) The CD absorbance spectra and (**B**) the 1st derivative spectra are displayed. The peaks are color coded.

RNA methylations have been reported to cause changes in the secondary structures of synthetic RNAs [51]. However, the effect of m^1^A and m^6^A marks on cellular RNAs have been poorly understood. Thus, we employed CD spectroscopy to probe the changes in the secondary structures of RNAs upon METTL3 and TRMT61A knockdown. Similar to the CD spectra of synthetic RNA molecules (Figure 3), the far-UV spectral region between 190 nm and 320 nm involves several strong/weak signals in the CD spectra of cellular total RNA samples. Accordingly, the CD spectra of control cellular RNAs exhibits a negative peak at 209.7 nm and three positive peaks at 198.7 nm, 224 nm (weak) and 265.8 nm (Figure 6A,B). Such CD spectral pattern corresponds to the forming of right-handed A-form of RNA conformation due to the sample dehydration during measurements. However, the amplitude and positions of those peaks are altered in the case of cellular total RNAs upon METTL3 or TRMT61A knockdown. Accordingly, the negative peak absorbing at 209.7 nm is reduced (almost half the amplitude), the positive peak at 198.7 nm is shifted down with reduced amplitude becoming less negative, and the weak CD signal at 224 nm becomes a less negative shoulder, reflecting a change in the helical twisting and RNA conformation due to reduced methylation. Additionally, a broad positive peak located between 240 and 300 nm is also detected with a maximum at 265.6 nm or 265.2 nm, becoming a less negative CD signal in the spectra of METTL3 or TRMT61A knockdown, respectively. Half of the the amplitude of this peak was reduced in TRMT61A knockdown total RNAs. Similar CD spectral alterations are also detected in the case of synthetic RNA oligonucleotides upon methylation (Figure 3). These altogether show that the dynamics, flexibility, base-stacking properties, and conformation of the RNA structure are altered in the presence of methylation, although all cellular total RNA samples probed here exhibit a right-handed A-form of RNA conformation. TRMT61A knockdown leads to more dramatic changes in comparison to METTL3 knockdown, even though their CD spectral profiles resemble each other (Figure 6).

### 3.3. TNF-α-Mediated Changes in the Biophysical Properties of RNAs

**Figure 7:**
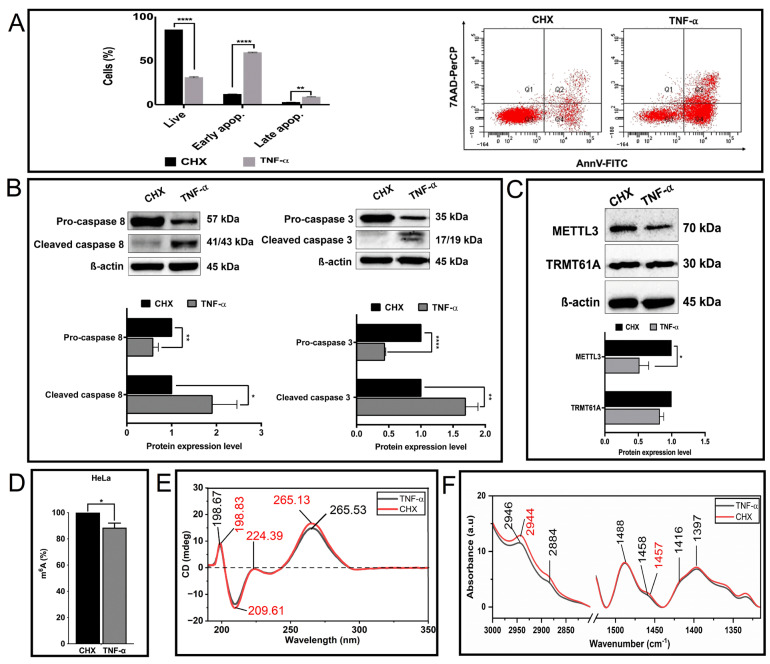
An analysis of the total RNAs from HeLa cells after CHX/TNF-α treatment. (**A**) Apoptosis rates of CHX/TNF-α treatment of HeLa cells. (**B**) Pro-caspase 3, Cleaved caspase 3, pro-caspase 8 and cleaved caspase 8 expression levels after CHX/TNF-α treatment. (**C**) METTL3 and TRMT61A expression levels after CHX/TNF-α treatment. (**D**) The change in the global m^6^A methylation amount after CHX/TNF-α treatment. (**E**) CD spectra and (**F**) FT-IR average absorbance spectra of total RNA from CHX/TNF-α treated HeLa cells. The peaks are color coded. Error bars indicate mean ± SD of three biological replicates. The statistical significance was demonstrated as * *p* < 0.05; ** *p* < 0.01; **** *p* < 0.0001 by two-tailed Student’s *t* test.

Encouraged by the distinctive property of FT-IR and CD spectroscopy in analyzing the global methylation status of total RNAs, we examined whether these approaches can be employed to examine perturbations in the cellular RNA methylome levels under physiological or pathological conditions. TNF-α is a ligand that primarily induces the extrinsic apoptotic pathway in HeLa cells [4,52]. We reported previously that TNF-α perturbs the expression of writer and eraser proteins concomitant with a distinct change in the m^6^A methylome in HeLa cells [4]. To uncover whether TNF-α treatment leads to any change in the global m^6^A amount, we induced apoptosis in HeLa cells by treating the cells with 37.5 ng/mL TNF-α or control CHX for 24 h. Flow cytometric and Western blot analyses showed that TNF-α induced early apoptosis in 59% of cells (Figure 7A,B). Interestingly, although TNF-α treatment caused 2-fold reduction in METTL3 amount compared to the control condition, we did not detect any statistically significant difference in TRMT61A amount (Figure 7C). We then exploited the colorimetric method to measure the total m^6^A methylation in total RNAs isolated from HeLa cells treated with TNF-α or control CHX for 24 h. Our data showed that TNF-α treatment causes 11.5% reduction in the total RNA m^6^A methylome (Figure 7D). We then performed FT-IR and CD analyses of total RNAs isolated from HeLa cells after CHX (control) and TNF-α treatment. We observed that both FT-IR and CD spectra exhibit a typical profile of A form RNA (Figure 7E,F), as detected in Figure 5 and Figure 6. The amplitudes of CD signals both at 265.5 nm and 209.6 nm are reduced and become less negative for total RNA isolated from TNF-α treated HeLa cells, indicating a decrease in the chirality of RNA molecules after TNF-α treatment when compared to control CHX treatment (Figure 7E). Additionally, the intensities of FT-IR signals in the spectral region of 3000–2800 cm^−1^ (due to CH_3_ stretching vibrations) and 1500–1300 cm^−1^ (due to CH_3_ bending vibrations) are slightly reduced after TNF-α treatment (Figure 7F). As TNF-α treatment induces a reduction in the total RNA m^6^A methylome when compared to control CHX treatment (Figure 7D), FT-IR and CD can clearly detect the minute alterations in the peak intensities and/or positions of CH_3_ groups of RNA methylation. These results are compatible with the colorimetric analysis of CHX/TNF-α treatment (Figure 7D) and are in good agreement with the FT-IR and CD analysis of cell-derived RNA samples from si-METTL3- and si-NC-transfected HeLa cells (Figure 5 and Figure 6).

## 4. Discussion

RNA methylation is emerging as an important layer of gene regulation that dictates the fate of both coding and noncoding transcripts [2]. Consequently, differential RNA methylation has been associated with numerous diseases [3,4,5,6]. In fact, RNA methylation has gained attention due to its potential implication in RNA therapeutics [9]. Therefore, efforts have been geared towards the development of detection methods for RNA methylation as well as understanding the impact of RNA methylation of RNA structure. To the best of our knowledge, so far there has been no report published on RNA methylation or any protocol that paves the way for the quantification of RNA methylation using FT-IR spectroscopy. In the current study, the methylation status of RNA molecules (cell-derived and synthetic oligonucleotides) was assessed with FT-IR and CD spectroscopies to explore the qualitative and quantitative spectral markers of m^6^A RNA and the effects on the RNA structures.

In the current study, an FT-IR analysis of un-methylated and m^6^A-methylated synthetic RNA samples showed that m^6^A RNA methylation induces unique spectral profiles (Figure 1 and Figure 2), successfully discriminating m^6^A moieties. m^6^A-methylated synthetic RNAs exhibit major spectral differences in the mid-IR region of 3000–2800 cm^−1^ (CH stretching vibrations) and 1500–1300 cm^−1^ (CH bending vibrations). Particularly, the IR signals absorbing at 2984 cm^−1^ and 1478 cm^−1^ due to the asymmetric stretching and bending vibrations of CH_3_ groups (methyl moiety) respectively are unequivocal indicators for the existence of methylation in RNA molecules. Significantly, when the methylation rate increases, those peaks linearly intensify with high correlations (R^2^: 0.98 for 2984 cm^−1^; R^2^: 0.96 for 1478 cm^−1^) (Figure 2). Thus, those pronounced IR signals absorbing 2984 cm^−1^ and 1478 cm^−1^ are the index of methylation and might be used for rapid and sensitive quantification of RNA methylation. Nevertheless, m^6^A-methylated synthetic RNAs exhibit IR peaks 2883 cm^−1^ and 1363 cm^−1^ due to the symmetric stretching and bending vibrations of methyl moiety, respectively (Figure 1 and Figure 2); however, those peaks have lower correlations with methylation concentration (R^2^: 0.76 for 2883 cm^−1^; R^2^: 0.66 for 1363 cm^−1^). Although the peak at 2984 cm^−1^ might be affected by spectral contributions of the O-H and N-H stretching vibrations of RNA molecule, this altogether violently proves that the intensities at 2984 cm^−1^ and 1478 cm^−1^ can be reliably used as intrinsic CH_3_ markers for the quantitative analysis of RNA methylation.

CD analysis reveals that synthetic m^6^A RNA oligonucleotides undergo minute alterations in the chirality and base-stacking properties stemming from methyl moiety when compared to un-methylated synthetic RNAs (Figure 3). The positive CD peak at 267 nm downshifts towards 266 nm and its amplitude increases, accompanied by an increase in the amplitude of negative peak at 209 nm for m^6^A-methylated synthetic RNAs (Figure 3). It was reported that the positive CD signal around 265 nm is affected by intra- and intermolecular interactions, as well as the base-stacking and base-pairing properties [24,31,45]. A recent study has also revealed that a reduction in the CD amplitude at 265 nm was found to be related to disruption of the base-stacking properties [24]. Additionally, the positive CD signals at 198 and 222 nm arise from the helical twisting of the RNA structure and are sensitive to base–base interactions and thus to the conformation of nucleic acids [24,53]. Nevertheless, in our work, no change was detected for the positive CD signal at 222 nm (Figure 3), indicating that the helical twisting of RNA was not affected upon m^6^A methylation, most likely due to having only one methyl group per one RNA nucleotide. Our results are in line with the findings reported in previous studies [29,32], in which m^6^A-induced slight changes or duplex–hairpin conversions in some RNA sequences have been reported, and the alterations in CD spectrum to the different extents due to m^6^A modifications in the RNA sequence were also shown. However, in our study, the hairpin formation was not observed (Figure 3).

Herein, FT-IR spectroscopy was also applied to determine the changes in the extent of total cellular RNA methylome levels upon METTL3 knockdown in HeLa cells. When compared to the total RNAs of control HeLa cells, the intensities of IR signals absorbing at 2949 cm^−^^1^ and 2883 cm^−^^1^ (the asymmetric and symmetric stretching vibrations of CH_3_ groups, respectively) as well as at 1488 cm^−^^1^ and 1359 cm^−^^1^ (the asymmetric and symmetric bending vibrations of CH_3_ groups, respectively) are strongly reduced upon METTL3 knockdown (Figure 5). This spectral pattern clearly reveals a reduction in the CH_3_ signals of methyl moiety in RNA molecules of METTL3 knockdown cells. Our data indicates that those IR signals (2949 cm^−^^1^, 2883 cm^−^^1^, 1488 cm^−^^1^ and 1359 cm^−^^1^) are the characteristic markers for RNA methylation, and thus, FT-IR can safely measure perturbations in the global m^6^A RNA marks upon METTL3 knockdown in eukaryotic cells. These analyses clearly suggest that FT-IR presents itself as a convenient and cheap method that can be safely used to measure the extent of global m^6^A RNA methylation from synthetic or cellular RNAs in response to stimuli. Subsequently, CD analysis also demonstrates the impact of METTL3 and TRMT61A knockdown on secondary structures of RNAs (Figure 6), revealing a change in the helical twisting and RNA conformation due to the attenuated methylation level. Interestingly, TRMT61A knockdown induces more pronounced alterations in RNA structures in comparison to METTL3 knockdown in HeLa cells.

To further support this, the same experimental approaches and measurement protocol were successfully used to detect a reduction in total RNA methylation upon treatment of HeLa cells with TNF-α. Based on the CD data, the chirality of total RNAs isolated from TNF-α-treated HeLa cells are altered upon reduction in the methylation level in comparison to the control CHX treatment (Figure 7E). CD analysis also shows that m^1^A and m^6^A methylation induce pretty distinct secondary structure on RNAs. When compared to control CHX treatment, FT-IR signals at 2946 cm^−1^ and 2884 cm^−1^ (CH_3_ stretching vibrations) and at 1488 and 1360 cm^−1^ (CH_3_ bending vibrations) are slightly decreased after TNF-α treatment (Figure 7F), revealing a smaller reduction in the total RNA m^6^A methylome. These results are in line with the colorimetric analysis (Figure 7D) and with the FT-IR and CD analysis of cell-derived RNA samples from HeLa si-METTL3 and HeLa si-NC (Figure 5 and Figure 6). Ultimately, we show that our protocol might be employed to identify perturbations in methylation marks upon extracellular stimuli in eukaryotic cells such as TNF-α, paving the way for its use in studying a cell- or condition-specific (for example, healthy versus cancer) analysis of RNA methylation.

FT-IR spectroscopy has many advantages for the analysis of RNA methylation. Molecular vibrations of functional groups in RNA simultaneously give rise to strong/weak IR signals in the mid-IR region (3600–800 cm^−1^). Our results showed that FT-IR analysis provides both qualitative and quantitative information on the RNA methylation. Additionally, the structural effects of methylation on the functional groups of RNA can be investigated, including conformational and dynamical alterations in the phosphodiester backbone, bases, sugar pucker, as well as helical transitions. Last but not least, we could successfully measure low RNA concentrations (up to 2 μg/mL) at low RNA volumes (2 μL, only one tiny drop), although FT-IR analysis is known to require a relatively high sample concentration. Herein, we used dried RNA samples (so-called dehydrated RNA) for FT-IR analysis to exclude the spectral contributions of free water molecules in the spectra. Since all RNA groups probed here are dehydrated with the same way, we can satisfactorily compare the RNA groups. Unless they are prepared with different drying procedure, the dehydrated samples of many biological specimens, such as cells, nucleic acids, (RNA, DNA) and body fluids, are commonly analyzed with FT-IR [13,16,19,44].

CD spectroscopy is advantageous for monitoring the structural alterations and conformational changes, and estimating the tertiary and secondary structures of biological samples at relatively low concentrations (nM, µM) and low volumes (10–700 µL) with minimal sample preparation procedures. [23,24,26,49,54]. The existence of base-stacking, helical, and loop structures in nucleic acids allows for the strong asymmetric structural change in RNA (or in DNA) detected between 185 nm and 350 nm, which corresponds to the π-π * transitions of bases and sugar-phosphate interactions, as well as to its n-π * transitions [24]. Our results showed that CD analysis allows us to figure out the structural changes in RNA due to the existence of the methyl group. Nevertheless, CD analysis has particular limitations in detection, such as quantitative information on RNA methylation marks.

In conclusion, we demonstrated a novel method/protocol based on FT-IR spectroscopy combined with CD analysis for qualitative and quantitative analysis of RNA methylation. The results of FT-IR spectra are in good agreement with CD experiments. Both FT-IR and CD data exhibit similar spectral profiles for both cell-derived RNA molecules and synthetic RNA oligonucleotides, revealing the minute changes in the RNA methylation status. m^6^A RNA methylation alters the RNA conformation which is sensitively detected in the FT-IR vibrational spectra and CD electronic absorption spectra through changes in the intensity and/or band position. The detection of concomitant IR signals of CH_3_ groups (vibrations of methyl moiety) in the FT-IR spectrum proves the existence of unequivocal indicators of methylation. Based on our FT-IR results, the intensities around 2949 cm^−1^ and 1488 cm^−1^, in particular, might be used to quantitatively determine the methylation in RNA samples isolated from cells. Although our data show that the 11.5% difference in RNA m^6^A methylation is easily detectable by FT-IR, more studies are required to determine the sensitivity of the protocol. Although our method provides valuable information about the global methylation state of cells, it does not provide information about the changes in a site-specific manner. The CD data reveal changes in chirality, base-stacking properties, and the conformation of RNA in the presence of methylation. Thus, FT-IR and CD spectroscopy as the rapid and label-free techniques can potentially be used to determine the methylation in cell-derived RNA samples and to identify the stacking configurations as well as secondary structures of RNA.

## Figures and Tables

**Table 1:** The sequences of oligomers used in the SELECT reactions.

Name	Sequence (5′ → 3′)
MALAT1 m^6^A2515 UP	tagccagtaccgtagtgcgtgAATTACTTCCGTTACGAAAG
MALAT1 m^6^A2515 DOWN	5phos/CCTTCACATTTTTCAAACTAAGCTACTcagaggctgagtcgctgcat
MALAT1 A2511 UP	tagccagtaccgtagtgcgtgAATTACTTCCGTTACGAAAGTCCT
MALAT1 A2511 DOWN	5phos/CACATTTTTCAAACTAAGCTACTcagaggctgagtcgctgcat
SELECT qRT-PCR Forward	ATGCAGCGACTCAGCCTCTG
SELECT qRT-PCR Reverse	TAGCCAGTACCGTAGTGCGTG

## Data Availability

Data are available upon request from the corresponding authors.

## Supplementary Materials

The following supporting information can be downloaded at: https://www.mdpi.com/article/10.3390/cells13221832/s1, Figure S1: The order of FT-IR data analysis procedure.
