# Supplementary material for: An Investigation of RNA Methylations with Biophysical Approaches in a Cervical Cancer Cell Model
Source: Cells. 2024 Nov 6;13(22):1832. doi: 10.3390/cells13221832 (PMC11592517; doi:10.3390/cells13221832)
Supplement: Supplementary file 1 [file cells-13-01832-s001.zip › cells-3226040-supplementary.pdf]

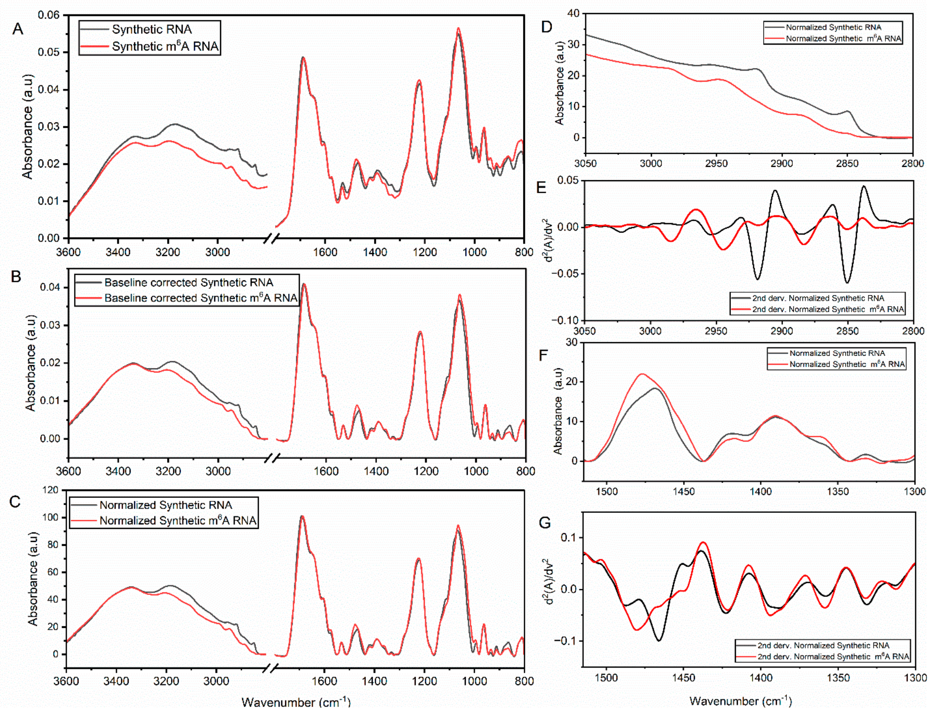

Supp. Figure S1. The order of FT-IR data analysis procedure. (**A**) Synthetic RNA raw data, (**B**) Baseline corrected data, (**C**) Normalized data (for equal area between 1750 cm^{-1} and 1513 cm^{-1}), (**D**) Zoom in for normalized data of CH stretching vibration region, (**E**) 2nd derivative of normalized data for CH stretching vibration region, (**F**) Zoom in for normalized data of CH bending vibration region, and (**G**), 2nd derivative of normalized data for CH bending vibration region. The FT-IR spectra were processed in order of panel A → panel B → panel C → panel D or panel F → panel E or panel G. The synthetic RNA samples without methylation (black) and with m6A methylation (red) are displayed.
